# Editorial: Investigating the molecular targets and therapeutic potential of Withania somnifera (Ashwagandha) in various pathophysiological conditions

**DOI:** 10.3389/fphar.2023.1187334

**Published:** 2023-05-02

**Authors:** Nawab John Dar, Shahnawaz Ali Bhat, Muneeb U. Rehman, Anthony Booker

**Affiliations:** ^1^ School of Medicine, UT Health, San Antonio, TX, United States; ^2^ Department of Zoology, FoLS, Aligarh Muslim University, Aligarh, India; ^3^ School of Pharmacy, King Saud University, Riyadh, Saudi Arabia; ^4^ School of Life Sciences, University of Westminster, London, United Kingdom

**Keywords:** Withania somnifera (Ashwagandha), neuroprotection, anti-cancer (anticancer) drugs, anti-infammatory activity, pharmacological action, natural products (NP), withanolides, withanolide (PubChem CID: 161671)

The therapeutic plant, Withania somnifera, commonly known as “Ashwagandha” or “Indian ginseng”, has been used for over 3,000 years in Indian Systems of Medicine for the treatment of various ailments. Known for promoting youthful vigor and enhancing muscle strength, endurance, and overall health, this plant contains over 50 chemical constituents with diverse biological implications. With preclinical studies demonstrating a broad range of pharmacologic properties, including anti-microbial, anti-inflammatory, anti-tumor, anti-stress, neuroprotective, and cardioprotective, Withania somnifera is a promising drug candidate for treating various clinical conditions, especially those related to the nervous system (Dar et al., 2015; Dar and Muzamil, 2020; Kashyap et al., 2022; Tewari et al., 2022). In this Research Topic, a comprehensive overview of the molecular targets of withanolides and the mechanistic basis of their action in various pathophysiological states has been presented. The Research Topic of research in this Research Topic highlights the therapeutic potential of Withania somnifera in cancer, neurodegenerative diseases, inflammatory and respiratory disorders.

The study by Singh et al demonstrates that Withania somnifera can be used as an add-on therapy for COPD patients. Chronic obstructive pulmonary disease (COPD) is a common and debilitating lung disease, and patients with COPD are at higher risk of severe outcomes from COVID-19. Current therapies for COPD lack disease-modifying potential and can cause adverse side effects. The study is a randomized, double-blind, placebo-controlled clinical trial involving 150 COPD patients. The results show that patients in the WS group have significantly improved lung function, quality of life, exercise tolerance, and reduced inflammation and oxidative stress compared to the placebo group. The phytochemicals in WS roots demonstrate potent inhibitory activity against ACE-2, MPO, and IL-6, and *in silico* studies show potential inhibitory activity against the SARS-CoV-2 receptor. These findings suggest that WS root may be a promising complementary medicine for COPD treatment, particularly in GOLD 2 and 3 categories of COPD patients.

The study by Bhat et al discusses the potential medicinal benefits of Withania somnifera in cancer and neurodegenerative diseases. The study suggests that Withania somnifera is effective in controlling disease progressions and could be a potential therapeutic target benefiting human health status. The study highlights the potential benefits of Withania somnifera in controlling disease progression and promoting DNA repair mechanisms, cellular repair, and preventing apoptosis of normal cells. Further, the study highlights the neuroprotective role of WS in several neurodegenerative conditions. It has demonstrated to reverse amyloid-induced toxicity, inhibit acetylcholinesterase activity, and increase the expression of neuroprotective protein in individuals with Alzheimer’s disease. In individuals with Parkinson’s disease, Withania somnifera treatment has provided dopaminergic neuroprotection and reduced oxidative stress. In individuals with Huntington’s disease, Withania somnifera has been shown to reduce neuronal damage and oxidative stress. The neuroprotective properties of Withania somnifera are thought to be due to its antioxidant and anti-inflammatory effects.

The study by Maushma Atteeq highlights the role of steroidal lactone (Withaferin A) derived from Withania somnifera. Withaferin A has been extensively studied for its anti-cancer properties, including interactions with key role players in cancerous activity that ultimately lead to cell death. Its pro-apoptotic properties include generating reactive oxidative species, activating Par-4, inducing endoplasmic reticulum stress, and activating p53. Withaferin A’s involvement in various oncogenic pathways leading to malignant neoplasm and its pharmacologic activity in conjunction with various cancer drugs provide promising evidence for its potential as a cancer treatment.

The study by Alanazi and Elfaki discusses that Withania somnifera possesses anti-inflammatory, antioxidant, anticancer, anti-diabetic, and anti-asthmatic properties. The therapeutic effects of Withania Somnifera are attributed to active ingredients such as withanolides, withaferins, and steroidal saponins. The current understanding of Withania Somnifera’s mechanisms of action is limited, but it has been proposed to promote cellular-mediated immunity or initiate chemical interactions contributing to therapeutic effects. The study focuses on Withania somnifera’s immunomodulatory effects and its role in treating immunological diseases by modulating cytokines, T-cell proliferation, and macrophage functions. The study also discusses Withania somnifera’s chemical properties and their immunomodulatory role in treating type 2 allergic diseases, where type 2 inflammation is imbalanced. Overall, the review suggests that Withania somnifera has promising therapeutic potential for treating various diseases through its immunomodulatory effects, and further research is needed to fully understand its mechanisms of action.

The study by Bashir et al discusses the potential therapeutic benefits of withanolides, a group of bioactive compounds found in Withania somnifera. The study reports that this medicinal plant is commonly used in Ayurvedic and indigenous systems of medicine. Various research groups have identified molecular targets of withanolides, including inhibiting the activation of nuclear factor kappa-B, promoting apoptosis of cancer cells, enhancing dopaminergic D2 receptor activity, and reducing the expression of the N-methyl-D-aspartate (NMDA) receptor. One of the active constituents, Withanolide-V may also have potential as an inhibitor of the main protease (Mpro) of SARS-CoV-2. However, the study suggests that these findings warrant further clinical trials to explore the potential therapeutic effects of withanolides on chronic diseases.

In conclusion, the current research indicates that Withania somnifera and its compounds possess significant therapeutic potential in various chronic diseases, particularly cancer and neurodegenerative conditions. Withania somnifera can serve as an adjunct therapy for COPD, and it also exhibits immunomodulatory properties. While the potential of Withania somnifera in treating COVID-19 requires further investigation, its potential as a treatment for chronic illnesses cannot be ignored. Further clinical trials are necessary to explore the full range of Withania somnifera’s therapeutic properties in chronic diseases.
